# A cross-sectional study of knowledge, attitudes, barriers and practices of cervical cancer screening among nurses in selected hospitals in the Eastern Cape Province, South Africa

**DOI:** 10.1186/s12905-023-02251-0

**Published:** 2023-03-09

**Authors:** Wezile Chitha, Siyabonga Sibulawa, Itumeleng Funani, Buyiswa Swartbooi, Kedibone Maake, Assegid Hellebo, Danleen Hongoro, Onke R. Mnyaka, Ziyanda Ngcobo, Christopher M. Zungu, Nomfuneko Sithole, Lizo Godlimpi, Sibusiso C. Nomatshila, Sikhumbuzo A. Mabunda, Vivien Essel

**Affiliations:** 1 Health Systems Enablement & Innovation Unit, University of the Witwatersrand, Johannesburg, South Africa; 2 Department of Public Health, Walter Sisulu University, Mthatha, South Africa; 3 School of Population Health, University of New South Wales, Sydney, Australia; 4 George Institute for Global Health, University of New South Wales, Sydney, Australia

**Keywords:** Cervical cancer, Screening, Nursing staff, KAP, Eastern Cape, South Africa

## Abstract

**Background:**

Cervical cancer is a preventable but highly prevalent cancer in many low -and middle-income countries including South Africa. Cervical cancer outcomes can be improved with improved vaccination, a well-coordinated and efficient screening programme, increased community awareness and uptake, and increased knowledge and advocacy of health professionals. This study therefore aimed to ascertain the knowledge, attitudes, practices and barriers of cervical cancer screening among nurses of selected rural hospitals in South Africa.

**Methods:**

A quantitative cross-sectional study was conducted in five hospitals in the Eastern Cape Province of South Africa between October and December 2021. A self-administered questionnaire was used to assess demographic characteristics of nurses and cervical cancer knowledge, attitudes, barriers and practices. A knowledge score of 65% was deemed adequate. Data were captured in Microsoft Excel Office 2016 and exported to STATA version 17.0 for analysis. Descriptive data analyses were used to report the results.

**Results:**

A total of 119 nurses participated in the study with just under two thirds (77/119, 64.7%) being professional nurses. Only 15.1% (18/119) of participants were assessed as having obtained a good knowledge score of ≥ 65%. The majority of these (16/18, 88.9%) were professional nurses. Of the participants with a good knowledge score, 61.1% (11/18) were from Nelson Mandela Academic Hospital, the only teaching hospital studied. Cervical cancer was deemed to be a disease of public health importance by 74.0% (88/119). However, only 27.7% (33/119) performed cervical cancer screening. Most of the participants (116/119, 97.5%) had an interest of attending more cervical cancer training.

**Conclusion:**

The majority of nurse participants did not have adequate knowledge about cervical cancer and screening, and few performed screening tests. Despite this, there is a high level of interest in being trained. Meeting these training needs is of utmost importance to implementing a comprehensive cervical cancer screening programme in South Africa.

## Background

Cervical cancer is most common in lower-income nations in sub-Saharan Africa, where 528 000 new cases are reported each year with low- and middle-income countries (LMICs) bearing more than 70% of the global burden [1–3]. Every year, millions of women throughout the world are diagnosed with cervical cancer, with more than half succumbing to the disease [1–4]. Although cervical cancer has been relatively well controlled in many high income countries (HICs), mainly because of cervical screening initiatives and effective cancer treatment services, it remains the most common cause of cancer related death among women in 42 countries including South Africa, most of which are LMICs [1, 5]. Cervical cancer awareness is generally low worldwide but worse in LMICs despite the increased prevalence of the disease [1, 6].

Sexually transmitted infections such as human immunodeficiency virus (HIV) and Human papillomavirus (HPV) are risk factors for female cancers [1, 7–9]. Cervical cancer is largely preventable through HPV vaccination, awareness, screening, medical outreach and early detection, and other preventative strategies such as protected sexual intercourse [1, 7–10]. HPV prevalence (low and high-risk) ranges between 44 and 85% among South African adolescents and young women (15–25 years) [8]. Both cervical cancer and HIV have a high prevalence in Africa [8]. The cervical cancer annual age-standardised rate (ASR) per 100 000 increased from 22.0 in 1998–2002 to 29.2 in 2008– 2012 in South Africa’s Eastern Cape Province [8]. HIV-infected women are at high risk of getting cervical intraepithelial neoplasia [11]. Many of these AIDS-defining cancers can threaten life as well as contribute to health problems [12].

In 2017, cervical cancer was reported to be the leading cancer in females in the OR Tambo and Alfred Nzo districts, Eastern Cape Province, South Africa [1, 13]. Cervical cancer rate in these two districts (18.8 per 100 000) is extremely high compared to the global average of 8.8 per 100 000 in 2018 [11]. Whereas cervical cancer is a preventable gynaecological cancer, the cancer progresses slowly in the lining of the cervix as precancerous lesions if not detected earlier or treated properly [14].

Cervical cancer is an ideal candidate for screening as it is asymptomatic in the early stages and has a long latent phase [15]. Cervical cancer screening detects precancerous cell transformations on the cervical mucosa that could progress to cervical cancer if not managed on time or appropriately [1, 14]. The Pap smear is a procedure which collects cells and mucus from the cervical mucosa and smeared onto the slide or a bottle of liquid and transported to the laboratory for examination [5]. Even though the 2017 South African guidelines allowed for the use of HPV-DNA testing as an alternative screening method (based on the availability of resources), cytology-based screening (Pap smear test) is still the more commonly used method in South Africa [16].

A majority of cancers, including cervical cancer are diagnosed at an advanced stage of disease because of lack of screening and early detection services, as well as limited awareness of early signs and symptoms of cancer [6]. Cytology-based screening is considered the best approach to reduce cervical cancer incidence in LMICs [1, 6]. According to the American Cancer Society guidelines for the early detection of cancer, Pap smear test should be started at the age of 21, regardless of sexual initiation or other risk factors [1, 16, 17].

HPV vaccination for primary school girls (presumably sexually naïve) is a key cervical cancer primary prevention strategy of South Africa’s cervical cancer policy [18]. This policy further advocates for the promotion of safe sexual practices and in that way hopes to reduce the HPV incidence [18]. Health workforce efficiency and competencies to screen, diagnose, and manage pre-cancerous cervical lesions are also matters prioritised by the policy [18]. The policy also places emphasis on strengthening of the service delivery platform by ensuring continuity of care through adequate referral pathways for women with a positive screen test result; resources (human and material) and infrastructure to treat women with positive screening results; and to build community awareness on service availability [18].

Through the public health sector, all South African women over the age of 30-years who are HIV negative and cervical cancer asymptomatic with normal screening results are entitled to three cervical cancer screening tests in ten year intervals [1, 18]. Furthermore, women living with HIV are deemed to be at high risk for cervical cancer regardless of their antiretroviral (ARV) treatment status [18]. As a result, in accordance with the policy, women living with HIV should be screened for cervical cancer every three years or annually (if the results are abnormal) for a lifetime irrespective of the CD4 count and ARV treatment status [18].

Colposcopy is a diagnostic process that allows for the detailed examination of the cellular patterns in the cervical epithelium and surrounding blood vessels [18]. This process enables the delineation of abnormal lesions and the subsequent biopsy of areas that are noted to be abnormal [18]. South Africa’s gold standard for abnormal cervical lesions is biopsy under colposcopy [18]. However, South Africa’s high prevalence of abnormal cervical pre-neoplasia and, the limited colposcopy trained health workers and shortages of colposcopy facilities makes it difficult to evaluate all women who deserve it [16].

Studies have shown that integrating knowledge and awareness programmes with educational interventions of cervical cancer screening will go a long way in early detection, reducing mortality and morbidity [1, 8, 15]. Furthermore, nurses are regarded as being a reliable source of health information in communities where they live [19]. Community members are not interested in the rank or department where the nurse might be working, as long as they know that a person is a nurse and they can easily access, they will ask questions that they find important to them [19].

It is important that health workers are educated, trained and well aware so that they can influence the beliefs and actions of the general public [7]. A 2006 study [20] conducted in other LMICs to measure the knowledge and awareness about cervical cancer screening amongst nurses found the knowledge of cervical cancer screening among nurses to be high while the uptake rate was abysmally poor [20]. A 2023 South African study [1] found primary care nurses to have poor knowledge on cervical cancer screening [1]. To our knowledge, there are no studies that have been undertaken in South Africa’s Eastern Cape Province to assess the knowledge and practices of cervical cancer screening amongst professional nurses in hospitals, probably because of the assumption that they already have adequate knowledge.

Negative attitudes of health workers, especially nurses can have a negative impact not only community responses to health programmes but can also impact health outcomes negatively [21, 22]. Negative attitudes can also have a direct association with adverse events[21, 22]. A 2022 study [22] in a hospital environment in Cyprus, associated the negative attitudes of doctors and nurses to being junior and being faced with a high workload. Furthermore, even though there are known patient barriers to poor programme uptake, negative attitudes of health workers contributes as one of the main barriers [21].

The knowledge, attitudes, practices and barriers are therefore important elements for designing, and monitoring strengths and challenges of screening programmes, as reported by nurses in the South African context [1, 6].

Health workers can play a fundamental part in raising awareness of the general public, and their knowledge needs to be assessed and updated on a regular basis [7]. Improving knowledge of the community members can improve their attitudes and at the same time potentially change their practices to seek healthcare early and embrace cervical cancer screening.

The study aimed to determine the knowledge, attitudes, barriers and practices of cervical cancer screening amongst nurses in selected hospitals in the Eastern Cape Province, South Africa. Hospitals have been chosen because cervical cancer screening and awareness is likely to be thought of as a primary care and/or gynaecology department responsibility. However, as stated above, women’s health is of critical public health importance and therefore of importance to the entire value chain, including hospitals and all departments.

## Methods

### Study design

This was a descriptive, quantitative cross-sectional study with analytical components conducted at five hospitals in the Eastern Cape Province of South Africa between the 18^th^ of October and 03^rd^ of December 2021. This study design allowed the researcher to survey multiple sites in a short space of time with no follow-up.

### Study setting

**Fig. 1:**
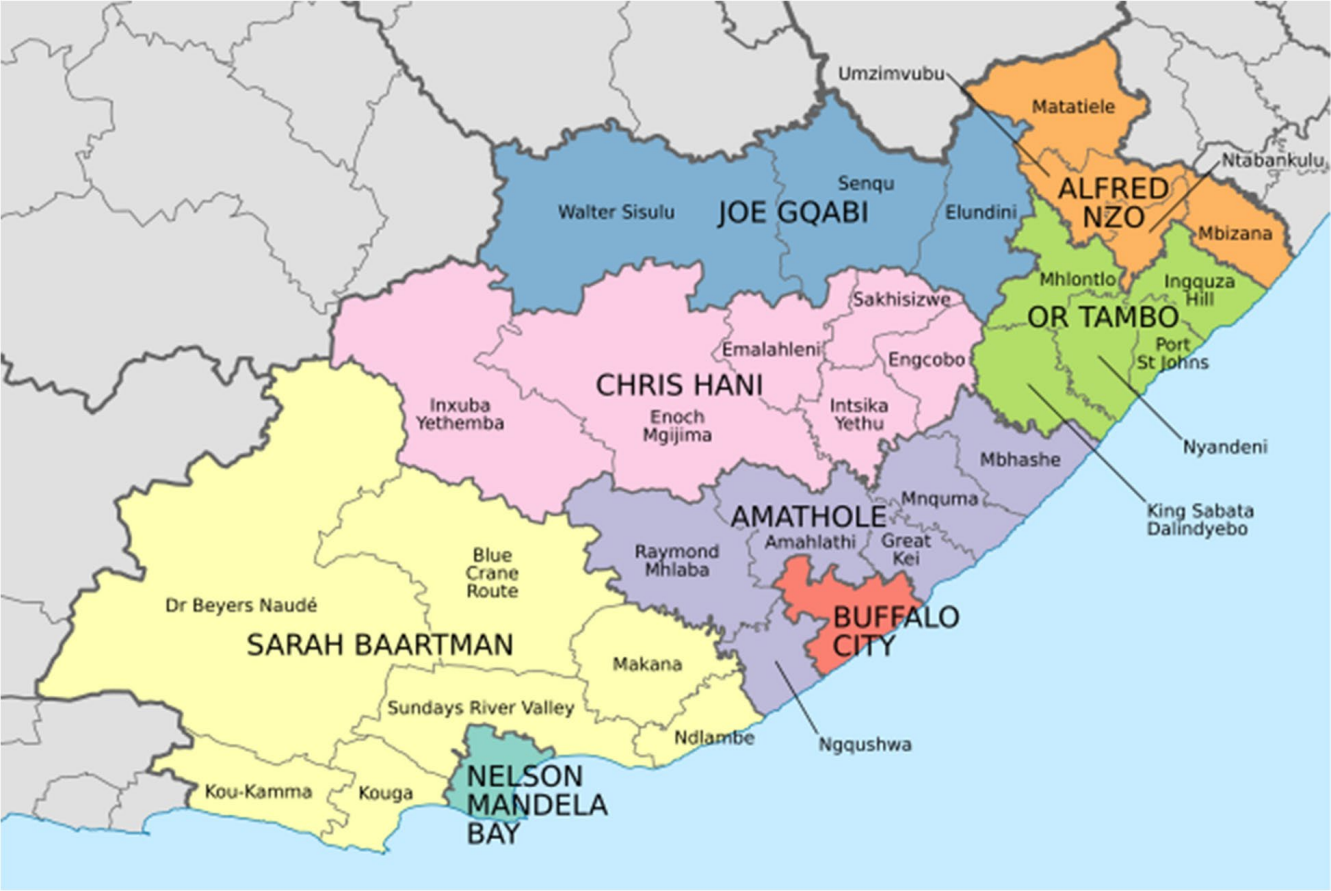
Map of the Eastern Cape Province showing the districts [23]

The Eastern Cape Province is in the South-Eastern part of South Africa. The province has eight health districts: Amathole, Chris Hani, Joe Gqabi, Sarah Baartman, O.R Tambo, Alfred Nzo as well as Nelson Mandela Metro and Buffalo City Metro [10], Fig. 1. More than 70% of the eastern part of the Eastern Cape live in rural areas [11]. The study only covers two districts (OR Tambo, and Alfred Nzo) that were conveniently selected due to their big population sizes and previous reports of high cervical cancer prevalence [13]. Five hospitals were randomly selected as study sites. A simple random sampling process selected St Elizabeth, St Patricks, St Barnabas, Madzikane kaZulu and Nelson Mandela Academic hospitals as study sites.

### Sample size and population

The study recruited professional and enrolled nurses who worked at any of the five sites. The sample size was calculated using the equation, $n=\frac{p\left(100-p\right){z}^{2}}{{d}^{2}}$ for a one-sided 95% confidence interval and a 5% significance level (z = 1.96). Because the proportion (p) of knowledge of nurses was not known, this (p) was set at 50% and the desired precision (d) was set at 10%. This thus yielded a minimum sample size of 96. To factor in for data entry errors, a further 20% (20) was added to yield a desired sample size of 116 participants for all five sites.

### Data collection

A self-administered validated quantitative data collection tool was used to determine the demographic characteristics of the respondents, and assessed respondents’ knowledge, attitudes, barriers and practices on cervical cancer screening. The latter questions were adopted from a standardised and validated tool for use in the community cancer screening outreach (BMSF Cancer Symptom Screening Tool, version 2.1; 27 August 2020). In addition, other questions were informed by prevailing literature themes from similar studies including one from Pakistan [24]. Two experts validated the final tool and piloted amongst five nurses in a different hospital from the study sites. To ensure anonymity between the researcher and the participants, the survey was conducted in a designated area of the health facility.

### Data management and analysis

Data were captured into Microsoft Excel Office 2016 and exported into STATA version 17.0 (STATA Corp, College Station, Texas, USA). Knowledge score of professional and enrolled nurses were assessed out of 100 and a knowledge score of 65% or more was deemed to be adequate. The Shapiro Wilk test was used to explore the distribution of numerical variables. Whilst normally distributed numerical variables were summarised using the mean, standard deviation (sd) and range, those that were not normally distributed were summarised using the median and interquartile range (IQR = 75^th^ percentile –(minus) 25^th^ percentile). Categorical variables were summarised using frequency tables, percentages and graphs.

Two or more categorical variables were summarised using contingency tables and the expected frequencies calculated to determine the type of test to use for the purpose of determining the extent of the relative associations. If the expected frequencies were ≥ 5, the Chi-squared test (Chi^2^) was used to compare two categories and if the expected frequencies were < 5, then the Fisher’s exact test was used. The Wilcoxon rank-sum test was used to compare medians of nursing categories if numerical data were not normally distributed and the two-sample t-test was used to compare the mean ages of participants between the two nursing categories as the age data was normally distributed. The 95% Confidence Interval (CI) was used to estimate the precision of estimates and the level of significance set at 5% (*p*-value ≤ 0.05) for statistical significance.

### Ethical considerations

The study obtained ethical clearance from the Human Research and Biosafety Ethics Committee of the Faculty of Health Sciences at Walter Sisulu University (040/2020) and from the University of the Witwatersrand Human Research Ethics Committee (M210211). Research access approval was obtained from the Eastern Cape Provincial Health Research Committees (EC_202010_012). Entry to the study sites was further negotiated with hospital management before data collection.

## Results

A total of 119 nurses participated in the study with just under two thirds (77/119, 64.7%) being professional nurses (Table 1). Of the total, 89.1% (106/119) were female. The average age of all participants was 41.8 years (*p*-value = 0.413), the youngest participant was 24-years old, the oldest was 62 years old, most participants (78/119, 65.5%) were between the age of 36 and 55 years and they had a median period of service of 6-years. Professional nurses had a median of 8-years in service (p25-p75 = 4–15 years); 27.3% (21/77) of professional nurses were in the outpatient department (OPD), 19.5% (15/77) were in the surgical ward and 15.6% (12/77) were in the gynaecology or oncology unit.

**Table 1:** Participants' demographic characteristics

Characteristics	Professional nurses		Enrolled nurses		Total	
Nursing category; n (%)	77	(64.7)	42	(35.3)	119	(100.0)
Age, years; mean ± sd (min–max)	42.4 ± 9.9	(24–62)	40.9 ± 8.2	(26–61)	41.8 ± 9.3	(24–62)
Age, years; n (%)						
≤ 35	21	(27.3)	12	(28.6)	33	(27.7)
36–45	20	(26.0)	15	(35.7)	35	(29.4)
46–55	29	(37.7)	14	(33.3)	43	(36.1)
56–62	7	(9.1)	1	(2.4)	8	(6.7)
Sex; n (%)						
Female	68	(88.3)	38	(90.5)	106	(89.1)
Male	9	(11.7)	4	(9.5)	13	(10.9)
Hospital; n (%)						
St Elizabeth	13	(16.9)	6	(14.3)	19	(16.0)
St Barnabas	16	(20.8)	10	(23.8)	26	(21.9)
St Patricks	13	(16.9)	11	(26.2)	24	(20.2)
Madzikane kaZulu	16	(20.8)	5	(11.9)	21	(17.7)
NMAH	19	(24.7)	10	(23.8)	29	(24.4)
Practice duration, years; median (p25 – p75)	8	(4–15)	4	(2–7)	6	(4–12)
Department; n (%)						
Gynaecology	7	(9.1)	1	(2.4)	8	(6.7)
Obstetrics	5	(6.5)	2	(4.8)	7	(5.9)
Oncology	5	(6.5)	1	(2.4)	6	(5.0)
OPD^#^	21	(27.3)	7	(16.7)	28	(23.5)
Emergency Unit	7	(9.1)	1	(2.4)	8	(6.7)
Infectious diseases	2	(2.6)	3	(7.1)	5	(4.2)
Surgical ward	15	(19.5)	12	(28.6)	27	(22.7)
Medical ward	4	(5.2)	7	(16.7)	11	(9.2)
High care ward	6	(7.8)	1	(2.4)	7	(5.9)
Paediatrics ward	5	(6.5)	7	(16.7)	12	(10.1)

^#^Outpatient department

p25 = 25^th^ percentile, p75 = 75^th^ percentile; *NMAH* Nelson Mandela Academic Hospital

**Fig. 2:**
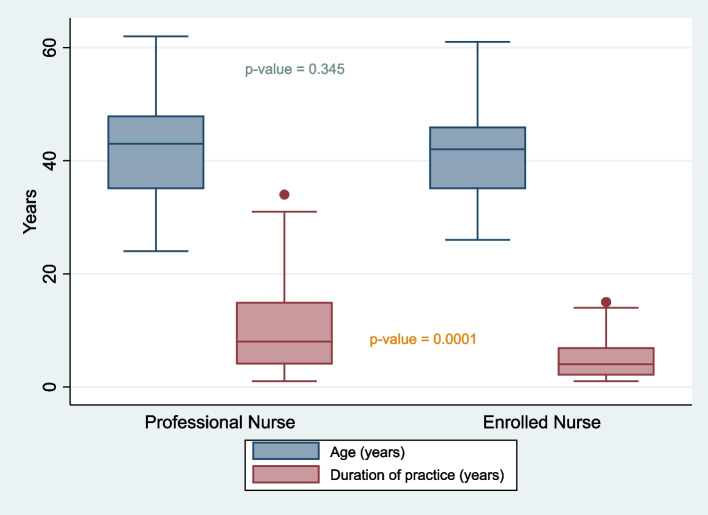
Duration of practice and age by occupational category

There was a statistically significant difference (*p*-value = 0.0001) between the duration of service of professional nurses and enrolled nurses (Fig. 2).

Only 15.1% (18/119) of participants were assessed as having obtained a good knowledge score of 65% and above (Table 2).

**Table 2:** Demographic associations of knowledge score

Demographic characteristics	Knowledge				
	**Good**		**Poor**		***p*-value**
Nursing category; n (%)					
Professional nurses	16	(88.9)	61	(60.4)	0.020
Enrolled nurses	2	(11.1)	40	(39.6)	
Age, years; n (%)					
≤ 35	3	(16.7)	30	(29.7)	0.239
36–45	6	(33.3)	29	(28.7)	
46–55	6	(33.3)	37	(36.6)	
56–62	3	(16.7)	5	(5.0)	
Hospital; n (%)					
St Elizabeth	1	(5.6)	18	(17.8)	0.004*
St Barnabas	1	(5.6)	25	(24.8)	
St Patricks	2	(11.1)	22	(21.8)	
Madzikane kaZulu	3	(16.7)	18	(17.8)	
NMAH	11	(61.1)	18	(17.8)	
Practice duration, years; median (p25 – p75)	10	(6–15)	5	(6–11)	0.017
Department; n (%)					
Gynaecology and Obstetrics	3	(16.7)	12	(11.8)	< 0.0001*
Oncology	5	(27.8)	1	(1.0)	
OPD^#^	5	(27.8)	23	(22.8)	
Paediatrics ward	1	(5.6)	11	(10.9)	
Other^~^	4	(22.2)	54	(53.5)	

^*^Fisher’s exact test was used

^#^Outpatient department; ^^^Other = divorced, separated and widowed

^~^Other = Emergency Unit, Infectious diseases unit, Surgical ward, Medical ward and High care ward; p25 = 25^th^ percentile, p75 = 75^th^ percentile; NMAH = Nelson Mandela Academic Hospital

Table 2 further shows that whilst only 20.8% (*n* = 16/77) of professional nurses had a good knowledge score, they accounted for 88.9% (*n* = 16/18) of the participants with a good knowledge score and this was statistically significant (*p*-value = 0.020). Of the participants with a good knowledge score, 61.1% (*n* = 11/18) were from Nelson Mandela Academic Hospital (NMAH) (*p*-value = 0.004); 27.8% each (*n* = 5/18) were from the oncology unit and outpatient department (*p*-value < 0.0001). In comparison to those with 5 or less years of practice, participants with good knowledge had a median duration of practice of 10 years or more (*p*-value = 0.017).

Cervical cancer was deemed to be a disease of public health importance by 74.0% (*n* = 88/119) of the participants, Table 3. Of the 106 female participants, 27.4% (*n* = 29) had not screened for cervical cancer before. Fourteen of the nurses (11.8%) did not like performing a pap smear and 19 (16.0%) felt that the cervical cancer vaccine should not be given to girls who were younger than 16 years. Most of the participants (*n* = 116/119, 97.5%) had an interest of attending more cervical cancer training.

**Table 3:** Knowledge and attitudes on cervical cancer

Measure	Professional nurses		Enrolled nurses		Total	
Cervical cancer is a disease of public health concern; n (%)						
Yes	62	(80.5)	26	(61.9)	88	(74.0)
No	15	(19.5)	16	(38.1)	31	(26.1)
Ever screened for cervical cancer^◊^; n (%)						
Yes	49	(72.1)	28	(73.7)	77	(72.6)
No	19	(27.9)	10	(26.3)	29	(27.4)
Do not like performing pap smear; n (%)						
Yes	6	(7.8)	8	(19.1)	14	(11.8)
No	71	(92.2)	34	(81.0)	105	(88.2)
Cervical cancer vaccine to not be given to younger than 16; n (%)						
Yes	9	(11.7)	10	(23.8)	19	(16.0)
No	68	(88.3)	32	(76.2)	100	(84.0)
Providers of cervical cancer screening**; n (%)						
Professional Nurses	10	(8.4)	5	(4.2)	14	(11.8)
Any Nurse	14	(11.8)	12	(10.1)	26	(21.8)
Enrolled Nurses	1	(0.8)	0	(0.0)	1	(0.8)
Any healthcare workers	35	(29.4)	12	(10.1)	47	(39.5)
Only health professionals	3	(2.5)	2	(1.7)	5	(4.2)
Medical doctors	8	(6.7)	8	(6.7)	7	(5.9)
Specialised Nurses	6	(5.0)	2	(1.7)	8	(6.7)
Gynaecologist	0	(0.0)	2	(1.7)	2	(1.7)
Other Health Workers	2	(1.7)	0	(0.0)	2	(1.7)
Cervical cancer training interest; n (%)						
Yes	75	(97.4)	41	(97.6)	116	(97.5)
No	2	(2.6)	1	(2.4)	3	(2.5)
Cervical examinations take too much time; n (%)						
Yes	8	(10.4)	7	(16.7)	15	(12.6)
No	69	(89.6)	35	(83.3)	104	(87.4)

^◊^ This only relates to 106 female participants

^**^ phrases were generated from open-ended responses overlaps between possible interpretations represent the understanding of participants

Only 25/77 (32.5%) of the professional nurses performed cervical cancer screening, 14/77 (18.2%) had received special training on cervical cancer screening and 10/77 (13.0%) had received special training on the interpretation of cervical screening results. Just over 11% (14/119, 11.8%) of the participants reported that some patients do not return to the hospital to confirm their results after being screened for cervical cancer (Table 4).

**Table 4:** Cervical cancer practices and experiences

Practices and experiences	Professional nurses		Enrolled nurses		Total	
Ever discussed cervical cancer screening with patients; n (%)						
Yes	56	(72.7)	21	(50.0)	77	(64.7)
No	21	(27.3)	21	(50.0)	42	(35.3)
Perform cervical cancer screening; n (%)						
Yes	25	(32.5)	8	(19.1)	33	(27.7)
No	51	(66.2)	34	(81.0)	85	(71.4)
No response	1	(1.3)	0	(0.0)	1	(0.8)
Frequency of patients who do not return for pap smear results; n (%)						
Very often	10	(13.0)	12	(28.6)	22	(18.5)
Often	19	(24.7)	6	(14.3)	25	(21.0)
Sometimes	38	(49.4)	20	(47.6)	58	(48.7)
Never	10	(13.0)	4	(9.5)	14	(11.8)
Ever referred patients for cervical screening; n (%)						
Yes	52	(67.5)	15	(35.7)	67	(56.3)
No	25	(32.5)	27	(64.3)	52	(43.7)
Special training on cervical cancer screening; n (%)						
Yes	14	(18.2)	4	(9.5)	18	(15.1)
No	63	(81.8)	38	(90.5)	101	(84.9)
Special training on interpretation of cervical screening results; n (%)						
Yes	10	(13.0)	5	(11.9)	15	(12.6)
No	67	(87.0)	37	(88.1)	104	(87.4)
Proportion of professional nurses not trained in cervical cancer screening; n (%)						
A lot	56	(72.7)	31	(73.8)	87	(73.1)
50%	13	(16.9)	1	(2.4)	14	(11.8)
10–50%	2	(2.6)	4	(9.5)	6	(5.0)
Less than 10%	3	(3.9)	3	(7.1)	6	(5.0)
None	3	(3.9)	3	(7.1)	6	(5.0)

**Fig. 3:**
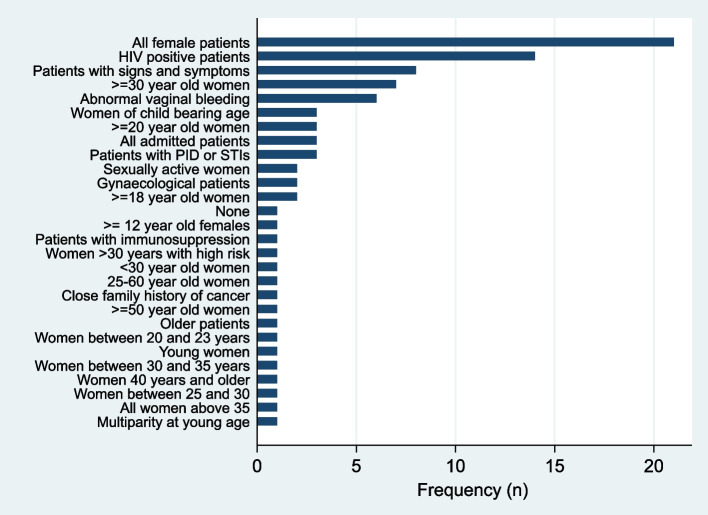
Patients who are offered cervical cancer screening

Whilst 21/119 (17.6%) of the participants reported to offer cervical screening to all female patients, 14/119 (11.8%) reported to offer it to HIV positive patients, 8/119 (6.7%) to patients with signs and symptoms, 7/119 (5.9%) to women who are 30 years and above, and 6/119 (5.0%) to women with abnormal vaginal bleeding (Fig. 3).

**Fig. 4:**
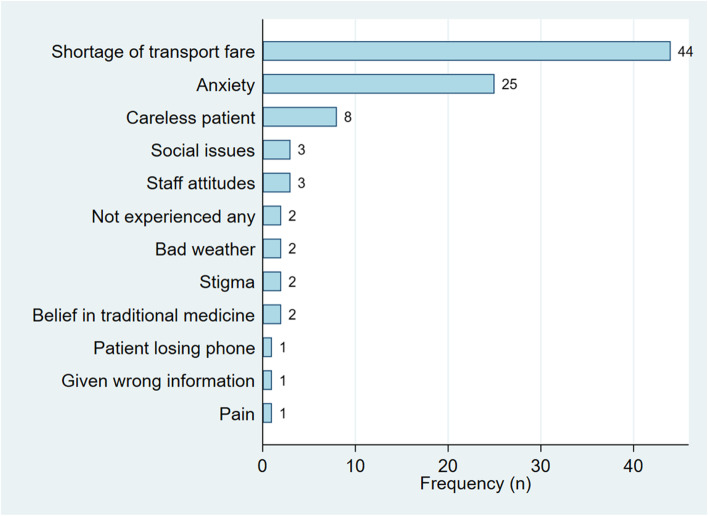
Cited reasons for patients who do not return for their cervical screening results

Reasons for patients to not return for their cervical screening results were reported to include shortage of transport fare (*n* = 44/119, 37.0%), anxiety about the possibility of a cancer diagnosis and/or cancer death (n = 25/119, 21.0%), careless or ignorant patient (*n* = 8/119, 6.7%), staff attitudes or social issues (*n* = 3/119, 2.5% each), and stigma, belief in traditional medicine or bad weather (*n* = 2, 1.7% each), Fig. 4.

## Discussion

In most health settings and communities, nurses are the more accessible, most trusted and largest single group of health professionals [25]. It therefore only makes sense to ensure that they are fully empowered on any public health intervention. This study sought to determine nurses’ knowledge, attitudes, barriers and practices on cervical cancer screening in five South African rural hospitals, including a teaching hospital. To the research team’s knowledge, this is the first study of its kind, to be conducted in a hospital setting in South Africa, and it will hopefully add to the existing body of knowledge on nurses’ non-compliance with cervical cancer screening guidelines and the poor cervical screening coverage by South African communities [26–28]. This study found nurses to have a limited knowledge on cervical cancer screening, some nurses did not consider cervical cancer to be of public health importance. A significant proportion of nurses who are eligible for cervical cancer screening had not yet been screened, 16.0% (19/119) of the participants were not comfortable with recommending the cervical cancer vaccine for girls who were younger than 16 years.

The fact that only 20.8% (16/77) of professional nurses had the adequate knowledge score of 65% suggests weaknesses in the health system. South Africa’s health system is predominantly nurse-based and requires nurses to have the competence (training and education) to perform cervical cancer screening and meet the community's health needs. A lack of skills and/or capacity development, such as nursing training and education, as well as resources in nursing, and shortages of nurses undermines and weakens nurses' ability to improve health outcomes and health system performance [29]. This study will therefore help facilitate discussions on the importance of a dedicated structure in place to implement the clinical education and training model for nurses to accelerate the implementation of cervical cancer screening programmes in South Africa, especially in rural areas. Furthermore, community uptake of HPV vaccines can only be improved if nurses and other health workers’ attitudes are improved as they will advocate for its uptake among community members. This study has therefore shown the need to help improve nurses’ attitudes towards the HPV vaccine and to help improve patients and health systems barriers to cervical cancer screening.

The proportion of nurses with good knowledge goes down further to 15.1% (18/119) when all the categories of nurses are considered. This figure contrasts that found in a study [30] conducted in India that showed that over 85% of health professionals had good knowledge of cervical cancer screening [30]. This study’s findings, are however, consistent to those of a primary care study in the same region in South Africa where only 53% of the nurses had adequate knowledge and only 53% of the nurses had adequate knowledge [1].

In this study, participants with higher cervical cancer screening knowledge are mostly those who worked in the teaching hospital (61.1%), those based in the oncology unit, gynaecology section or OPD, and those with a median duration of practice of ten years. These findings suggest rationed exposure of knowledge on matters of public health importance such as cervical cancer screening. First, whilst it would be unfair to expect nurses who do not practice on a subject on a daily basis to have the same amount of knowledge as those who do, there are at least some basics that they must know especially considering the duration of the cervical cancer screening policy in South Africa. More so, cervical cancer is not just a primary care problem but a broader women’s health and public health issue. Capacity building of nurses should therefore have such considerations to ensure better health outcomes for society.

Second, if the policy is not well understood by nurses, regardless of their practice role, by inference it will be poorly understood by community members. This therefore questions the policy translation processes, advocacy attempts and empowerment of health workers who are not necessarily practicing in the area of cervical cancer screening. The findings largely mirror those of other policies, such as a lack of organisational competencies to implement care delivery models that shift care from the hospital to the community, and ineffective monitoring, governance, training, and education when it comes to implementing a comprehensive cervical cancer screening programme [31].

It is of concern that more than a quarter of the participants did not view cervical cancer as a disease of public health importance. More so because the participants are nurses and mostly female. This negative attitude is revealed further in the 27.4% of eligible nurse participants who had not been screened for cervical cancer and the nurses who were not comfortable with a cervical cancer vaccine. It is however encouraging that the majority (97.5%) were interested in a cervical cancer training. Efforts have to therefore be undertaken to broaden the exposure of some information (including but not limited to cervical cancer) to all categories of nurses regardless of their practice specialty or placement. These could start off with reviewal of the nursing curriculum even before they choose or are allocated to a practice specialty. These training programmes and curricula sometimes need innovation such as the flipped classroom model for nurses described elsewhere in literature [32].

The fact that 11.8% of nursing staff reported that some patients do not return to the hospital to confirm their results after being screened for cervical cancer suggests that it is very common for patients to not return for results after being screened. Patients should be made to understand the reasons for cervical cancer screening in a clear language. Detailed counselling should be provided after screening, and a system to remind patients of their return dates for screening results should be instituted. Community health workers should be asked to trace patients who do not return for screening results on scheduled date.

Even though minimised, this study had limitations inherent to an observational study that uses a self-administered questionnaire. First, only nurses who worked day shifts were sampled due to the bigger proportion of day staff and the limited number of staff who work at night and weekends who would be unlikely to cooperate due to work pressures. There is, however, no reason to believe that night and weekend staff would have responded any different to these participants. Second, the use of a self-administered questionnaire could have resulted in participant dishonesty as there was no means of validating their responses. This was mitigated through the assurance of participants before commencement that their responses would be anonymous and that they could withdraw whenever they were not comfortable. Third, the sample size is small to generalise to all nurses in South Africa or elsewhere outside the study context. Fourth, the use of a survey did not allow for probing of responses. This study, however, forms a good basis for future exploratory studies on improving policy implementation, ensure compliance and improve its impact and reach.

## Conclusion

Cervical cancer is a preventable cancer that can be detected early through screening. It can also be prevented through HPV vaccination, early diagnosis and early treatment. Nurses are key to the goals of improving the prognosis of cervical cancer survivors. This study has exposed the poor knowledge of nurses on cervical cancer in four South African rural hospitals. This study has also revealed negative attitudes of nurses towards HPV vaccine and screening, issues the health system needs to address urgently. Several patient and health systems barriers affect the uptake of cervical cancer screening such as the reportedly high numbers of patients who do not return to health facilities for their results after screening. All these challenges need a systemic approach to nurse education, capacity building of nurses, health systems strengthening and nurse curriculum development and reviews.

## Data Availability

The data that support the findings of this study are available from the corresponding author upon reasonable request.

## Abbreviations

ARV: Antiretroviral
BMSF: Bristol myers squibb foundation
HIC: High income country
HIV: Human immunodeficiency virus
HPV: Human papillomavirus
LMIC: Low- and middle-income countries
NMAH: Nelson Mandela Academic Hospital
OPD: Outpatient department
